# COVID‐19: medical students in clinical research

**DOI:** 10.1111/tct.13241

**Published:** 2020-08-27

**Authors:** Scarlet‐Daisy Prior, Tamsin McKinnon, Victoria Gresty, Max Mulligan, Liam Richards, Alastair Watson, Christopher A Green

**Affiliations:** ^1^ Birmingham Medical School University of Birmingham Birmingham UK; ^2^ Clinical and Experimental Sciences Faculty of Medicine University of Southampton Southampton UK; ^3^ NIHR Southampton Biomedical Research Centre Southampton Centre for Biomedical Research Southampton General Hospital Southampton UK; ^4^ Senior Clinical Lecturer in Infectious Disease Institute of Microbiology and Infection University of Birmingham Birmingham UK

1

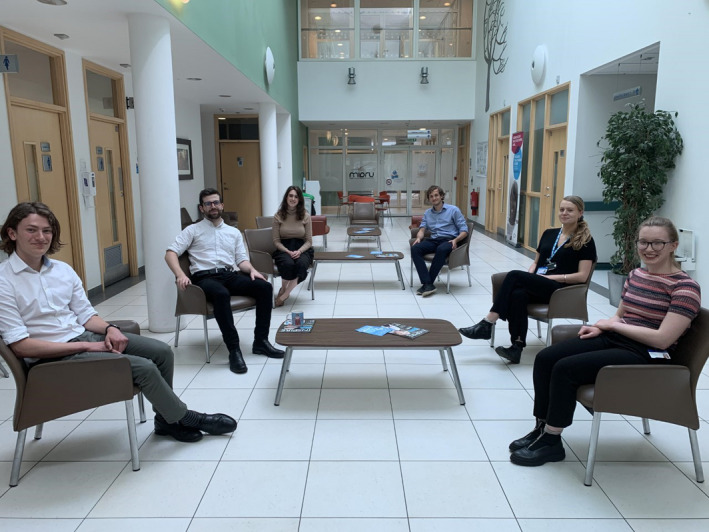

In response to the COVID‐19 pandemic, thousands of clinical research staff from across the UK were redeployed to the front line of the National Health Service (NHS).^1^ This left a depleted workforce to deliver the urgent public health research (UPHR) projects required to characterise the novel disease, inform public health measures and evaluate potential therapeutics. As medical students at the University of Birmingham, we received an urgent appeal from Dr Chris Green (co‐author) and the National Institute of Health Research (NIHR) to volunteer to help deliver these clinical studies. We responded with the aim to support the NIHR and NHS workforce, and positively contribute to the unfolding crisis.^2^ We further sought to experience clinical research first‐hand as, despite coming from a range of backgrounds, we had few insights into the practicalities of organising and conducting clinical trials.

Our volunteer roles have varied in response to emerging research needs. As part of the International Severe Acute Respiratory and Emerging Infections Consortium (ISARIC), we collected serial data on COVID‐19 inpatients’ symptoms and observations.^3^ Our role later expanded to assisting with the Randomised Evaluation of COVID‐19 Therapy (RECOVERY) trial to help screen patients for eligibility, assess their capacity to consent and explain the trial to patients, alongside and under the supervision of local NHS research and development (R&D) staff.

Our experiences have not only supported UPHR delivery but have also highlighted the learning opportunities that clinical research presents to medical students. The need for evidence‐based medicine is reinforced throughout our medical training; however, with limited access to research during medical school, contextualising this concept is difficult. During our experience, we have observed the demand for a COVID‐19 therapeutic agent and the process required to demonstrate its safety and efficacy. We have also been able to appreciate the uncertainty inherent in both research and clinical practice. Whereas the patient cases presented during our education often have clearly defined answers and learning objectives, we have seen that outcomes for individual patients and clinical trials are not as well defined. This is a more nuanced view, which should serve us well as future doctors.

It is difficult for medical schools to prepare students fully for the sudden responsibility that we will experience as newly qualified doctors; however, we believe that clinical research placements could play a role in doing this. Whilst volunteering, we have been responsible for organising our own work schedule and presenting our ideas and findings to the wider research team. We have also rapidly learned to implement our theoretical understanding of concepts such as critical appraisal, communication, mental capacity assessment and taking informed consent. Furthermore, for many of us, our first patient consultations have not been the typical, highly supported, semi‐artificial scenarios, but an exchange of critical information relating to a global pandemic. We have developed an improved understanding of how scientific data are collected, which will enhance our ability to assess available evidence critically and make erudite clinical decisions in the future.^4^

The newly developed NIHR clinical research volunteer network is more representative of the multidisciplinary teams found within the NHS. As students at different stages of training and with varying levels of experience, we have had the chance to interact across year groups, having had limited opportunity to do so otherwise in medical school. Opportunities to discuss our ideas and questions about the studies with consultants and the wider clinical research team have also given us unique insights into the current understanding of COVID‐19, potential treatments and vaccines. These opportunities and the research network that we have developed will be invaluable to help us engage with future research and inform our career choices.^5^

The research experience available to us during the COVID‐19 pandemic has been invaluable to our professional development. Given the requirement for high levels of advanced training and expertise, however, opportunities to participate in research during medical school are currently limited. This seems counterintuitive, as many of us will play critical roles in future clinical research. We believe opportunities for clinical research placements should therefore be incorporated into medical education. These placements should be coupled with Good Clinical Practice (GCP) training for medical students, to ensure both compliance with ethical practices and the safety of patients.^6^ Although we recognise mandatory placements and training could stretch an already busy medical curriculum, and may not be welcomed by all students, we believe that it will benefit our understanding and engagement with evidence‐based medicine, clinical trials and researcher–clinician collaborations. This will help with the effective delivery of future clinical research and will ultimately benefit our patients.

The research experience available to us during the COVID‐19 pandemic has been invaluable to our professional development

… opportunities for clinical research placements should therefore be incorporated into medical education
